# Hyperprogression after Immune Checkpoint Inhibitors: A Cloudy Phenomenon with Real-life Consequences

**DOI:** 10.34133/cancomm.0035

**Published:** 2026-06-17

**Authors:** Damien Bruyat, Nicolas Girard, Pierre Heudel, Nicolas Penel, Marie-Cécile Le Deley, Issaga Diallo, Jean-Marie Boher, Christophe Zemmour, Brice Chanez, Anne Madroszyk, Gwenaelle Gravis, Emilien Billon, Aaron Lisberg, Bérénice Collineau, Mathilde Beaufils, Anthony Gonçalves, Manuel Tejeda, Anne-Sophie Chrétien, Philippe Rochigneux

**Affiliations:** ^1^ Department of Medical Oncology, Institut Paoli-Calmettes, Marseille, France.; ^2^ Aix-Marseille Université, INSERM U1068, CNRS UMR7258, Centre de Recherche en Cancérologie de Marseille (CRCM), Immunity and Cancer Team, Marseille, France.; ^3^ Department of Medical Oncology, Institut Curie, Paris, France.; ^4^ Department of Medical Oncology, Centre Léon Bérard, Lyon, France.; ^5^ Department of Medical Oncology, Centre Oscar-Lambret, Lille, France.; ^6^ ULR 2694–METRICS (Évaluation des technologies de santé et des pratiques médicales), Centre Hospitalo-Universitaire de Lille, University of Lille, Lille, France.; ^7^ Directorate of Clinical Research and Innovation, Institut Paoli-Calmettes, Marseille, France.; ^8^ Department of Medical Oncology, David Geffen School of Medicine at University of California Los Angeles (UCLA), Los Angeles, CA, USA.

The advent of immune checkpoint inhibitors (ICIs) has revolutionized the management of solid tumors, improving overall survival (OS) [1]. However, some patients experience an unexpected acceleration of tumor growth after ICI initiation, exceeding the natural course of the disease. This phenomenon, called “hyperprogressive disease” (HPD), was described in 2017 by Champiat et al. in a cohort of 218 patients treated in phase I trials with anti-programmed death ligand 1 (PD-L1) or programmed cell death protein 1 (PD-1) inhibitors. Among the 131 evaluable patients, 12 (9%) were identified with HPD, defined as a ≥2-fold increase in tumor growth rate during anti-PD-1/PD-L1 therapy compared with the pretreatment period. These “hyperprogressors” had a 26-fold increased risk of death in multivariable Cox analysis adjusted for the Royal Marsden Score [2]. In 2017, Saâda-Bouzid et al., using an alternative mathematical definition of HPD, reported a prevalence of 29% in a cohort of 34 patients with head and neck cancer treated in phase II trials, with no substantial impact on OS [3]. Heterogeneous definitions and conflicting data complicate hyperprogression diagnosis in routine practice. Variability in prevalence and survival impact reflects a limited understanding, with no consensus for clinical use [4]. Data outside trials remain scarce, and better recognition of HPD in daily practice is needed to guide therapeutic decisions, including ICI discontinuation and consideration of rescue chemotherapy [5]. The primary objective of the present study (HYPERPROG study) was to identify real-life HPD under ICIs as detected by oncologists, to characterize their clinical and biological profiles (Supplementary Materials).

The HYPERPROG study is a retrospective, multicentric study, supported by the Institut Paoli-Calmettes (Marseille, France). Between 2017 January 1 and 2023 September 30, across 4 French cancer centers, 13,250 patients with solid tumors received at least 1 infusion of ICI (Fig. S1). Among them, 100 medical records mentioned “hyperprogression”, identified using the ConSoRe natural language processing tool, and 57 met the selected definition for HPD (tumor growth rate ratio >2 + clinical deterioration). We excluded equivocal cases with alternative diagnoses (e.g., immunoallergic pneumonitis or dissociated response). We also excluded 2 cases of radiological HPD without clinical deterioration, as their disease trajectory differed markedly from the rest of the cohort. These exclusions ensured a clinically meaningful and homogeneous HPD population.

The number of HPD cases reported per center and the most common primary tumor types are summarized in Table S1. According to our identification strategy, clinicians’ notes mentioned HPD cases in 0.43% (57/13,250) of patients, with a range between 0.16% and 0.89% depending on the center. The most common primary tumor sites associated with HPD were the lung (36.84%), kidney (14.03%), and head and neck (14.03%; Table S2). Preferential sites of HPD (i.e., organs in which new lesions appeared and/or existing lesions progressed) stratified by primary tumor type are shown in Fig. 1A. For lung cancer, hyperprogression was predominantly locoregional (20/21, 95.24%). In head and neck tumors, HPD was mainly local (6/8, 75.00%), whereas in kidney cancer, it was frequently distant with pleuropulmonary involvement (5/8, 62.50%). Overall, the lung was the most frequent site of hyperprogression.

**Fig. 1.**
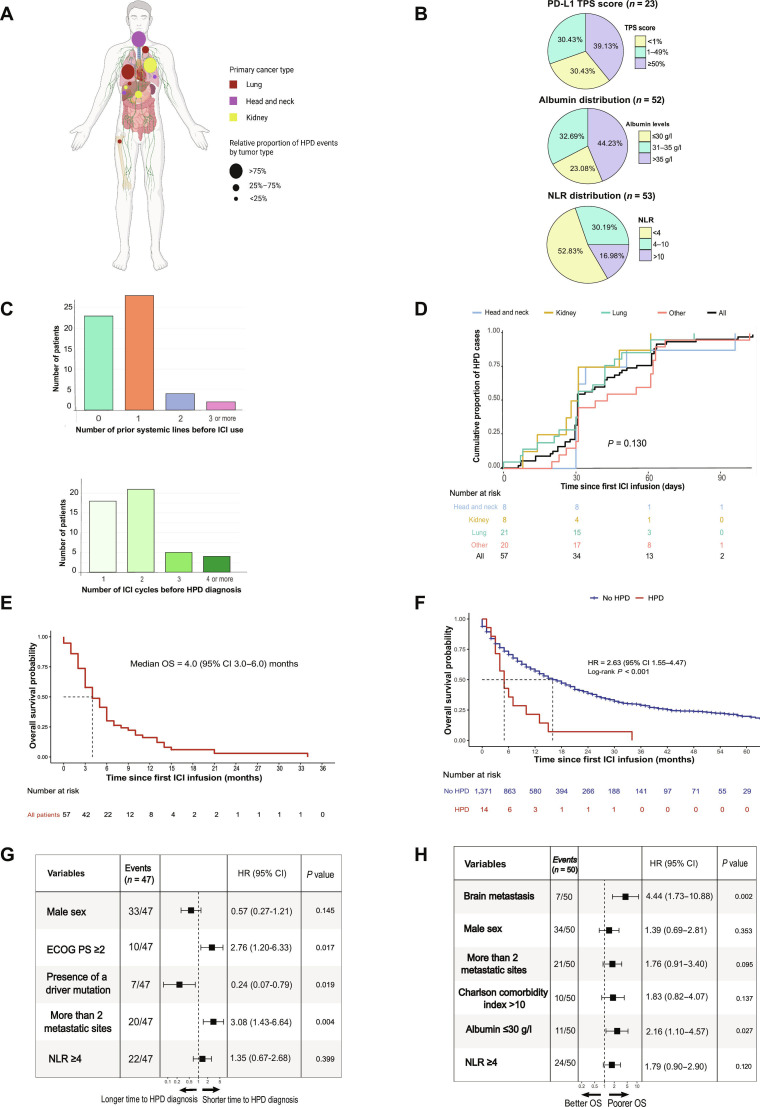
Clinical and biological characteristics of hyperprogressive disease (HPD) in solid tumor patients treated with immune checkpoint inhibitors (ICIs) in the multicentric HYPERPROG study (*n* = 57). (A) Distribution of HPD sites stratified by primary tumor type (lung, kidney, and head and neck cancers). Circles represent the relative proportion of HPD events occurring at each anatomical site for each primary tumor subgroup. Circle sizes are scaled and categorized (<25%, 25% to 75%, and >75%) separately for each primary tumor type and do not reflect overall HPD incidence. This figure was created with BioRender.com. (B) Distribution of key baseline biological characteristics in patients with HPD, including programmed death ligand 1 (PD-L1) tumor proportion score (TPS) score, serum albumin level, and neutrophil-to-lymphocyte ratio (NLR). (C) Top: Number of prior systemic therapy lines before ICI initiation (*n* = 57). Bottom: Number of ICI cycles before HPD onset (*n* = 48). In both panels, the y-axis represents the number of patients. (D) Time from ICI initiation to HPD diagnosis among patients with confirmed HPD, overall and according to primary tumor type. Curves were plotted using 1 − *S*(*t*) derived from Kaplan–Meier estimates. Median time to HPD was 31 (95% confidence interval [CI] 31 to 42) days. The reported *P* value was obtained using an exploratory log-rank test. (E) Overall survival (OS) of patients with HPD (*n* = 57) was estimated using the Kaplan–Meier method. (F) Comparison of OS between patients with HPD (red, *n* = 14) and patients without HPD (blue, *n* = 1,371) treated with ICIs in the Institut Paoli-Calmettes cohort. The non-HPD cohort includes patients with complete response, partial response, stable disease, and standard progressive disease. Survival curves were compared using the log-rank test. (G) Variables associated with time to HPD diagnosis were identified using multivariable Cox proportional hazards regression. (H) Variables associated with OS were identified using multivariable Cox proportional hazards regression. Hazard ratios (HRs) are presented with respective 95% CIs. Statistical significance was defined as *P* < 0.05. Multivariable analyses were performed on available cases (complete-case analysis).

Herein, patients with HPD were predominantly male (38/57, 66.67%), with a median age of 64 years (Table S3). They had a good general condition before ICI initiation: 70.18% (40/57) had an Eastern Cooperative Oncology Group Performance Status of 0 to 1 and relatively few comorbidities (median Charlson Comorbidity Index of 9). Most patients had metastatic disease (89.47%, 51/57), while 6 patients (10.53%) had locally advanced disease. In the overall cohort, 27 patients (47.37%) had limited metastatic involvement (1 to 2 sites), and 24 (42.11%) had extensive involvement (>2 sites). No patients were treated in the neoadjuvant setting. Pembrolizumab and nivolumab were the most used ICIs in the present study. Most patients (89.47%, 51/57) received ICIs without chemotherapy, and only 5 (8.77%) had an ICI combination (ipilimumab plus nivolumab). Immune-related adverse events (any grades) occurred in 10 patients (17.54%), including 3 cases of interstitial pneumonitis (Table S4). Interestingly, PD-L1 tumor proportion score was equally distributed across the categories (Chi-square goodness-of-fit test, *P* = 0.842; Fig. 1B). Regarding nutritional status, 23.08% (12/52) of patients had low albumin serum levels (≤30 g/l). For the neutrophil-to-lymphocyte ratio (NLR), 52.83% (28/53) had a NLR <4, 30.19% (16/53) had NLR 4 to 10, and 16.98% (9/53) had NLR >10.

Among patients with HPD, 40.35% (23/57) received ICIs as first-line therapy, 28 (49.12%) as second-line therapy, 4 (7.02%) as third-line therapy, and 2 (3.51%) in the fourth-line or later (Fig. 1C). Hyperprogression predominantly occurred during the early ICI cycles, with 18 patients (31.58%) developing HPD after cycle 1 and 21 patients (36.84%) after cycle 2. In the combination therapy group, with the exception of 1 missing data point, all cases of hyperprogression occurred during the first 2 cycles of ICIs (Table S5).

The median time to hyperprogression was 31 (95% confidence interval [CI] 31 to 42) days (Fig. 1D) with 77.19% (44/57) occurring within the first 2 months*.* In this descriptive analysis restricted to patients with confirmed HPD, the distribution of time from ICI initiation to HPD diagnosis did not show clear differences across primary tumor types (exploratory log-rank *P* = 0.130). In the overall population, median OS was 4.0 (95% CI 3.0 to 6.0) months, and the 12-month OS rate was 19% (Fig. 1E). The combination-therapy subgroup exhibited a trend toward worse OS compared to the monotherapy subgroup (hazard ratio [HR] = 2.05, 95% CI 0.80 to 5.23; log-rank *P =* 0.135) (Fig. S2). In an independent cohort of patients treated with ICIs from Institut Paoli-Calmettes (*n* = 1,385) [6], patients with HPD (*n* = 14) had a median OS of 5.0 (95% CI 4.0 to 15.0) months versus 16.0 (95% CI 15.0 to 19.0) months in those without HPD, with a significantly increased mortality risk (HR = 2.63, 95% CI 1.55 to 4.47; log-rank *P* < 0.001; Fig. 1F). However, the “non-HPD” group included patients with complete remission, partial remission, stable disease, and standard disease progression; therefore, it is not possible to distinguish between the survival of patients with HPD and that of patients who would have progressed without meeting the HPD criteria.

We then sought to determine, among patients with confirmed HPD, which factors were associated with earlier HPD diagnosis after ICI initiation, as well as with OS. Univariable Cox analyses are presented in Tables S6 and S7. In our multivariable analysis (Fig. 1G and Fig. S3), the presence of a driver mutation was associated with a longer time to HPD diagnosis (HR = 0.24, 95% CI 0.07 to 0.79; *P* = 0.019). In contrast, Eastern Cooperative Oncology Group Performance Status ≥2 and high tumor burden (>2 metastatic sites) were independently associated with a shorter time to HPD diagnosis (HR = 2.76, 95% CI 1.20 to 6.33; *P* = 0.017 and HR = 3.08, 95% CI 1.43 to 6.64; *P* = 0.004, respectively). In the multivariable analysis of OS (Fig. 1H and Fig. S3), brain metastases were associated with worse survival (HR = 4.44, 95% CI 1.73 to 10.88; *P* = 0.002), as was serum albumin level ≤30 g/l (HR = 2.16, 95% CI 1.10 to 4.57; *P* = 0.027).

Through one of the largest cohorts of hyperprogressive patients described to date, the HYPERPROG study characterized the clinical and biological patterns of hyperprogression. Our clinician-reported case ascertainment, which likely underestimates HPD, was not designed to assess incidence or risk factors, unlike systematic reviews and meta-analyses [7]. Instead, our aim was to describe real-world HPD—its speed, severity, and organ involvement—to enable early identification and adapted management of high-risk patients, including early computed tomography reassessment (4 to 6 weeks after ICI initiation) and consideration of chemotherapy or anti-vascular endothelial growth factor-based approaches [5]. In our cohort, higher tumor burden and poor performance status were associated with earlier HPD onset, whereas low serum albumin at ICI initiation was associated with poorer survival after HPD. Overall, HPD emerged as an uncommon yet early and clinically important pattern of progression.

## Ethical Approval

The HYPERPROG study was conducted in accordance with the Declaration of Helsinki and applicable French regulations. The study protocol was reviewed and approved by the institutional review board of Institut Paoli-Calmettes (protocol reference: HYPER-PROG-IPC 2022-002, approval date 2022 January 24). Given the retrospective, noninterventional nature of the study and the use of anonymized data extracted from electronic medical records, informed consent was waived in accordance with French legislation. Data processing complied with applicable data protection regulations.

## Data Availability

The datasets generated and/or analyzed during the current study are not publicly available due to data protection regulations and institutional policies but are available from the corresponding authors upon reasonable request and subject to appropriate data-sharing agreements.
