# Supplementary material for: Hyperprogression after Immune Checkpoint Inhibitors: A Cloudy Phenomenon with Real-life Consequences
Source: Cancer Commun (Lond). 2026 Jun 17;46:0035. doi: 10.34133/cancomm.0035 (PMC13273061; doi:10.34133/cancomm.0035)
Supplement: Supplementary 1 — Figs. S1 to S3 Tables S1 to S7 [file cancomm.0035.f1.docx]

**Supplementary Materials for**

**Hyperprogression after Immune Checkpoint Inhibitors: A Cloudy Phenomenon with Real-life Consequences.**

Damien Bruyat^1, 2, *^, Nicolas Girard^3^, Pierre Heudel^4^, Nicolas Penel^5, 6^, Marie Cécile Le Deley^6^, Issaga Diallo^6^, Jean Marie Boher^7^, Christophe Zemmour^7^, Brice Chanez^1^, Anne Madroszyk^1^, Gwenaelle Gravis^1^, Emilien Billon^1, 2^, Aaron Lisberg^8^, Bérénice Collineau^1^, Mathilde Beaufils^1^, Anthony Gonçalves^1^, Manuel Tejeda^6^, Anne Sophie Chrétien^2^, and Philippe Rochigneux^1, 2, *^

^1^ Department of Medical Oncology, Institut Paoli-Calmettes, Marseille, France.

^2^ Aix-Marseille Université, INSERM U1068, CNRS UMR7258, Centre de Recherche en Cancérologie de Marseille (CRCM), Immunity and Cancer Team, Marseille, France.

^3^ Department of Medical Oncology, Institut Curie, Paris, France.

^4^ Department of Medical Oncology, Centre Léon Bérard, Lyon, France.

^5^ Department of Medical Oncology, Centre Oscar-Lambret, Lille, France.

^6^ ULR 2694–METRICS (Évaluation des technologies de santé et des pratiques médicales), Centre Hospitalo-Universitaire de Lille, University of Lille, Lille, France.

^7^ Directorate of Clinical Research and Innovation, Institut Paoli-Calmettes, Marseille, France.

^8^ Department of Medical Oncology, David Geffen School of Medicine at University of California Los Angeles (UCLA), Los Angeles, California, USA.

*Address correspondence to:

P.R. (Philippe Rochigneux), [rochigneuxp@ipc.unicancer.fr](mailto:rochigneuxp@ipc.unicancer.fr); D. (Damien Bruyat), [damien.bruyat@etu.univ-amu.fr](mailto:damien.bruyat@etu.univ-amu.fr)

**Supplementary Methods**

***Study Design***

HYPERPROG-study is a retrospective, multicentric study, supported by the Institut Paoli-Calmettes (Marseille, France), designed to describe hyperprogression in patients treated using immune checkpoint inhibitors (ICIs) between January 1, 2017 and September 30, 2023. This study is based on ConSoRe technology [1], a powerful data mining software that extracts information from patients’ medical records. This technology is used by French cancer centers; among them, 4 participated in the study: Institut Paoli-Calmettes (IPC, Marseille), Institut Curie (IC, Paris), Centre Léon Bérard (CLB, Lyon), Centre Oscar Lambret (COL, Lille). Patients were treated according to each center’s usual practices in a real-life setting. Our institution's Review Board accepted the protocol (HYPER-PROG-IPC 2022-002) on 01/24/2022. As this was a retrospective, non-interventional multicenter study based on anonymized data and without impact on patient management, no additional separate IRB approval numbers were issued by the other participating centers. To facilitate collaboration and ensure data safety, data-sharing agreements were established with all participating centers. Patients’ data were anonymized in compliance with applicable privacy regulations.

***Extracting electronic patient records with the ConSoRe tool***

ConSoRe is an academic data analytics solution developed by UNICANCER (a network of 18 French Comprehensive Cancer Centers) in collaboration with Intel and Sword Group. ConSoRe performs data mining by aggregating structured and unstructured data from Electronic Patient Records (EPR) using Natural Language Processing (NLP) [1]. Using ConSoRe, we built a cohort of patients with solid tumors that received ICIs (alone or in combination – chemotherapy, tyrosine kinase inhibitors, other ICIs) in our 4 centers, by screening for the comprehensive list of all ICIs routinely available in France in our timeframe: “atezolizumab”; “durvalumab”, “ipilimumab”, “nivolumab”, “pembrolizumab” (Query 1). Then we extracted patients with mention of hyperprogression in the EPR using an AND operator (Query 2). The search was submitted to ConSoRe as follows, based on correspondence to at least one keyword (see below). After this selection, patients’ data were manually extracted from the EPR.

- Query 1: *[« Atezolizumab », « Durvalumab », « Ipilimumab », « Nivolumab », « Pembrolizumab »].* This search was limited to pharmacy databases; that is, we selected patients who had an ICIs administered and validated by a cancer center pharmacist. This avoided cases where ICIs were considered but never administered.
- Query 2: *[« Hyperprogression », « Hyper progression », « hyperprogress », and «hyperprogressor»].* The aim was to identify the term "hyperprogression" wherever it appeared in the patient's records.

***Hyperprogression definition***

To check the authenticity of hyperprogression, we used the definition proposed by Champiat et al. [2] as follows: Equal to or more than a 2-fold increase in tumor growth rate (TGR) between pre-ICIs scan and first re-evaluation scan. TGR was calculated on the assumption that tumor growth is exponential, with the following formula:

$$TGR=100\times{(Exp}^{(TG)}-1)$$

Where: $TG=\frac{3 \times\log\frac{T2}{T1}}{Time}$

Abbreviations: TG, Tumor Growth; TGR, Tumor Growth Rate; T1, tumor size at pre-ICIs scan; T2, tumor size at first re-evaluation scan; Exp, Exponential; Time (in months) = (date 2 – date 1) / 30.44. With 30.44 corresponding to the mean number of days in a month annually.

To help discriminate hyperprogression from pseudoprogression or “standard” progression (the principal differential diagnoses), we associated the notion of clinical deterioration [3] . Therefore, patients had to show clinical deterioration, assessed by a decrease in Performance Status (PS), defined as a decline of at least one point on the Eastern Cooperative Oncology Group (ECOG) scale, and/or the occurrence of organ failure (grade ≥3 according to Common Terminology Criteria for Adverse Events v4.0)

***Inclusion and exclusion criteria***

Patients were included if they presented the following criteria: i) having a pathologically confirmed diagnosis of solid tumor; ii) receiving ICI between January the 1^st^ 2017 and September 30, 2023; iii) presenting at least one relevant keyword in the EPR for each Query; iii) meeting the predefined criteria for hyperprogression. Conversely, patients were excluded in case of insufficient medical data, lack of imaging to confirm hyperprogressive disease (HPD) diagnosis, or if an alternative diagnosis was more likely to explain the observed progression (immunoallergic response, dissociated response, progression under ICI, hyperprogression under tyrosine kinase inhibitor, and pseudoprogression).

***Variables of interest***

To precisely describe the HPD population, we collected variables of interest from the EPR. Biological variables (Lactate dehydrogenase (LDH) level, PD-L1 expression according to Tumor proportion score (TPS), and albumin level) or clinical variables (such as cancer location, liver metastasis, metastatic site number, previous treatment line, and type of ICIs) were chosen on the basis of the review by Liu [4], Kim [5], and Zhao et al. [6]. To evaluate patients' comorbidities, we used the Charlson Comorbidity Index (CCI), a prognostic score validated in longitudinal studies, particularly in cancer patient populations [7-9]. We used the same threshold for neutrophil-lymphocyte ratio (NLR) as in the study by Zer et al. [10]. The albumin threshold was set at 30 g/L, in line with the definition of severe undernutrition proposed by the French health authority. The biological values entered into the database were those closest to the first day of treatment with ICI, from one month before ICI to two weeks after ICIs were administered either as monotherapy or as combination immunotherapy. Monotherapy included anti–PD-1 agents (pembrolizumab, nivolumab, and durvalumab) and anti–PD-L1 therapy (atezolizumab), and could be given alone or with chemotherapy. Combination immunotherapy consisted exclusively of ipilimumab plus nivolumab and was always administered without chemotherapy.

***Outcomes definition***

Overall survival (OS) was defined as the duration from the first dose of ICI to death or last follow-up, with no restriction on the cause of death [11]. Death status and death date were checked in the publicly available French death register(https://deces.matchid.io/search). Follow-up was defined as the time from the first ICI dose to censoring. Cumulative incidence of HPD was defined by the time from the first ICI to HPD occurrence. Time to HPD onset was defined as the time between the first ICI dose and the diagnosis of hyperprogression. The median time to HPD was estimated using the Kaplan–Meier method and corresponded to the time point at which the cumulative incidence reached 50%.

***External cohort data***

To evaluate a potential detrimental OS data in patients with HPD versus patients without HPD treated with ICI, we used a retrospective cohort of solid tumor patients treated with ICI at the Institut Paoli-Calmettes (*n* = 1,385) [12]. This cohort, published by Beaufils et al., was focused on ICI-induced dysthyroidism (*n* = 90), but also included hyperprogressive patients (*n* = 14) identified in our HYPERPROG study. We compared OS between hyperprogressive patients and the rest of the cohort as part of a complementary analysis. Detailed treatment response categories (i.e., CR, complete response; PR, partial response; SD, stable disease; and PD, progressive disease) were not available, and analyses were therefore limited to survival outcomes.

***Statistical analysis***

Population characteristics were reported using both absolute values and percentages. Descriptive statistics were used to characterize the study population, using counts and frequencies for categorical endpoints and medians (ranges) for quantitative variables; Chi-squared tests assessed associations between categorical variables. Time to HPD was studied as a continuous outcome, in days. Time to HPD was analyzed using the Kaplan–Meier method. The median time to HPD corresponded to the time at which the estimated survival probability reached 0.5. The proportion of HPD events over time was estimated using the Kaplan–Meier method, and the cumulative event curve was displayed as 1 − S(t). Univariate and multivariate Cox proportional hazards models were used to assess the prognostic value of the variables of interest for HPD incidence and overall survival. Among patients with confirmed HPD, time to HPD diagnosis was analyzed as a time-to-event outcome. Univariate and multivariate Cox proportional hazards models were used to explore associations between baseline variables and the timing of HPD onset. These analyses were descriptive and should not be interpreted as estimating the risk of developing HPD in an unselected ICI-treated population. All variables with a *P* value < 0.2 in univariate analysis were included in multivariate Cox models, presented in tables and forest plots. Multivariable Cox analyses were performed using available data for each variable. Patients with missing values were excluded on a per-variable basis, except for driver mutation status, which was included as a categorical variable with three levels (“alteration detected”, “no alteration detected”, and “unknown” when next-generation sequencing was not performed), allowing retention of patients with missing molecular data in the model. Here, “driver alteration” refers to a known oncogenic molecular alteration involved in tumorigenesis and potentially targetable in routine practice, depending on tumor type; seven patients had a driver mutation: four with *EGFR* mutations (no further details provided), *KRAS G12D*, *BAP1*, and *BRAF V600E*. The proportional hazards ratio (HR) assumptions for these variables were verified using Schoenfeld residuals tests, ensuring the validity of the Cox models. Survival curves were estimated using the Kaplan-Meier method (survminer R package, version 0.5.0) and compared using two-sided log-rank tests. All statistical analyses were performed using R statistical software (version 4.3.2). All comparisons were two-sided, with *P* < 0.05 being considered significant.

**Supplementary Tables**

**Supplementary Table S1. Number of HPD cases reported per clinician notes according to each center.**

| **Cancer center (City)** | **Number of HPD reported per clinician notes/Total of patients treated using ICIs (%)** | **Most common primary tumor type, *n* (%)** |
| --- | --- | --- |
| Institut Curie (Paris) | 22/4,310 (0.51%) | Lung, 10 (45.45%) |
| Institut Paoli-Calmettes (Marseille) | 14/1,580 (0.89%) | Lung, 8 (57.14%) |
| Centre Oscar Lambret (Lille) | 13/2,262 (0.57%) | Kidney, 4 (30.77%) |
| Centre Leon Bérard (Lyon) | 8/5,098 (0.16%) | Head and neck, 3 (37.50%) |

Abbreviations: HPD, hyperprogressive disease; ICI, immune checkpoint inhibitor

**Supplementary Table S2. Primary cancer location in the HYPERPROG study (*n* = 57).**

| **Primary cancer location** | **Number of patients, *n* (%)** |
| --- | --- |
| Lung | 21 (36.84) |
| Kidney | 8 (14.03) |
| Head and neck | 8 (14.03) |
| Melanoma | 6 (10.53) |
| Urothelial | 5 (8.77) |
| Breast | 2 (3.51) |
| Colorectal | 1 (1.75) |
| Cholangiocarcinoma | 1 (1.75) |
| Anal canal | 1 (1.75) |
| Mesothelioma | 1 (1.75) |
| Soft tissue sarcoma | 1 (1.75) |
| Vulva | 1 (1.75) |
| Unknown | 1 (1.75) |

**Supplementary Table S3. Characteristics of patients with HPD (*n* = 57)**

| **Characteristics** | **Patients with HPD** |
| --- | --- |
| Sex, *n* (%) | |
| Male | 38 (66.67) |
| Female | 19 (33.33) |
| Age at diagnosis, median (range), years | 64 (28-90) |
| ECOG PS before ICI use, *n* (%) | |
| 0 | 16 (28.07) |
| 1 | 24 (42.11) |
| 2 | 9 (15.79) |
| 3 | 1 (1.75) |
| NA | 7 (12.28) |
| Charlson Comorbidity Index at diagnosis, median (range) | 9 (2-14) |
| Driver mutation, *n* (%) | |
| Alteration detected | 7 (12.28) |
| No alteration detected | 18 (31.58) |
| Unknown | 32 (56.14) |
| Metastasis, *n* (%) |  |
| Yes | 51 (89.47) |
| No | 6 (10.53) |
| Metastasis site before ICI infusion, *n* (%) |  |
| Liver | 20 (39.22) |
| Brain | 9 (17.65) |
| Other | 22 (43.13) |
| Number of metastatic sites, *n* (%) |  |
| 0 | 6 (10.53) |
| 1-2 | 27 (47.37) |
| >2 | 24 (42.11) |
| Concomitant chemotherapy^a^, *n* (%) |  |
| Yes | 6 (10.53) |
| No | 51 (89.47) |
| Immune checkpoint inhibitor, *n* (%) |  |
| Pembrolizumab | 24 (42.11) |
| Nivolumab | 20 (35.09) |
| Ipilimumab + Nivolumab | 5 (8.76) |
| Atezolizumab | 4 (7.02) |
| Durvalumab | 4 (7.02) |
| Immune-related adverse events (any grades), *n* (%) | 10 (17.54) |
| Radiological confirmation of HPD, *n* (%)^b^ |  |
| CT | 47 (82.45) |
| MRI | 6 (10.53) |
| PET | 5 (8.77) |
| Laboratory test, median (range)^c^ |  |
| Albumin, g/L, | 33.5 (20-46) |
| LDH, UI/L, | 302 (154-1771) |
| NLR, | 3.8 (0.8-54.5) |

^a^ 3 patients received chemotherapy plus pembrolizumab, 2 received chemotherapy plus durvalumab, and 1 patient received chemotherapy plus atezolizumab

^b^ One patient was diagnosed with HPD based on both a CT scan with contrast and a liver MRI

^c^ Biochemical data at the start of ICI treatment were not available for all patients: albumin (*n* = 52), LDH (*n* = 36), and NLR (*n* = 53).

Abbreviations: ECOG PS, Eastern Cooperative Oncology Group Performance Status; NA, not available; ICI, immune checkpoint inhibitor; HPD, hyperprogressive disease; CT, computed tomography; MRI, magnetic resonance imaging; PET, positron emission tomography; LDH, lactate dehydrogenase; NLR, neutrophil-to-lymphocyte ratio.

| **Adverse events** | **Number of events** |
| --- | --- |
| Interstitial pneumonitis | 3 |
| Bronchial spasticity | 1 |
| Mucositis | 2 |
| Hepatitis | 2 |
| Xerosis | 2 |
| Arthralgia | 2 |
| Diarrhea | 1 |

**Supplementary Table S4. Immune-related adverse events.**

A total of 13 adverse events were reported in 10 patients: one patient experienced mucositis and diarrhea, one patient had dry skin and joint pain, and one patient had mucositis and hepatitis.

**Supplementary Table S5. Clinical and biological characteristics of patients with hyperprogressive disease according to ICI regimen**

| **Characteristics** | **Overall (*n* = 57)** | **Monotherapy^a^ (*n* = 52)** | **Combination therapy^b^ (*n* = 5)** | ***P* value** |
| --- | --- | --- | --- | --- |
| Age at diagnosis, median (IQR), years | 64 (55-72) | 65 (56-72) | 50 (49-71) | 0.303 |
| CCI, median (IQR) | 9 (7-10) | 8 (7-10) | 10 (9-10) | 0.298 |
| irAEs, *n* (%) | 10 (17.54) | 10 (19.23) | 0 (0.00) | 0.574 |
| ECOG PS before ICIs, *n* (%) |  |  |  | 0.503 |
| 0 | 16 (28.07) | 13 (25.00) | 3 (60.00) |  |
| 1 | 24 (42.11) | 22 (42.31) | 2 (40.00) |  |
| 2 | 9 (15.79) | 9 (17.31) | 0 (0.00) |  |
| 3 | 1 (1.75) | 1 (1.92) | 0 (0.00) |  |
| NA | 7 (12.28) | 7 (13.46) | 0 (0.00) |  |
| Number of metastatic sites >2, *n* (%) | 24 (42.11) | 20 (38.46) | 4 (80.00) | 0.151 |
| Serum albumin level <30 g/L, *n* (%)^c^ | 12 (23.08) | 12 (23.08) | 0 (0.00) | 0.578 |
| NLR >4, *n* (%)^d^ | 25 (47.17) | 22 (42.31) | 3 (60.00) | 0.658 |
| Number of ICI infusion before HPD diagnosis, *n* (%) |  |  |  | 0.711 |
| 1 | 18 (31.58) | 15 (28.85) | 3 (60.0) |  |
| 2 | 21 (36.84) | 20 (38.46) | 1 (20.00) |  |
| 3 | 5 (8.77) | 5 (8.77) | 0 (0.00) |  |
| 4 | 2 (3.51) | 2 (3.85) | 0 (0.00) |  |
| 5 | 1 (1.75) | 1 (1.75) | 0 (0.00) |  |
| 6 | 1 (1.75) | 1 (1.75) | 0 (0.00) |  |
| NA | 9 (15.79) | 8 (15.38) | 1 (20.00) |  |

^a^ Monotherapy = Pembrolizumab or Nivolumab or Atezolizumab or Durvalumab

^b^ Combination therapy = Ipilimumab plus Nivolumab

^c^ Available Data for Serum albumin level: *n* = 52

^d^ Available data for NLR: *n* = 53

Abbreviations: irAEs, Immune-related adverse events; IQR, interquartile range; CCI, Charlson comorbidity index; ECOG PS, Eastern Cooperative Oncology Group Performance Status; NLR, neutrophil-to-lymphocyte ratio; ICI, immune checkpoint inhibitor; NA, not available,

**Supplementary Table S6. Univariable analyses of factors associated with time from first ICI infusion to HPD diagnosis.**

| **Variables**^a^ | **Univariate analysis, HR (95% CI)** | ***P* value**^b^ |
| --- | --- | --- |
| Male sex | 1.54 (0.87-2.71) | 0.140 |
| Age ≥65 | 0.85 (0.50-1.44) | 0.540 |
| ECOG PS ≥ 2 | 2.33 (1.12-4.81) | 0.024 |
| CCI >10 | 1.04 (0.55-1.99) | 0.890 |
| Driver mutation | 0.51 (0.23-1.14) | 0.100 |
| Metastatic disease | 0.98 (0.41-2.34) | 0.963 |
| Brain metastasis | 1.22 (0.59-2.52) | 0.590 |
| Liver metastasis | 1.04 (0.60-1.80) | 0.900 |
| Concomitant chemotherapy | 1.19 (0.50-2.79) | 0.700 |
| Level of PD-L1 expression | 0.99 (0.55-1.77) | 0.970 |
| Type of ICI^c^ | 1.10 (0.51-2.37) | 0.810 |
| Adverse events | 0.61 (0.29-1.27) | 0.200 |
| Albumin ≤30 g/L | 0.84 (0.44-1.62) | 0.600 |
| Metastatic sites >2 | 1.62 (0.93-2.84) | 0.089 |
| LDH >300 UI/L | 0.91 (0.47-1.78) | 0.890 |
| NLR ≥4 | 1.69 (0.95-2.98) | 0.073 |

^a^ Univariable analyses were conducted in the overall HPD cohort; the number of evaluable patients varied according to data availability for each variable.

^b^ Only variables with a *P* value < 0.2 were included in the multivariate analysis.

^c^ Refers to the following categories: anti-PD-1 inhibitors, anti-PD-L1 inhibitors, or combination therapy consisting of anti-CTLA-4 plus anti-PD-1/PD-L1 inhibitors.

Abbreviations: HPD, hyperprogressive disease; CI, confidence interval; HR, hazard ratio; NE, not evaluated; ECOG PS, Eastern Cooperative Oncology Group Performance Status; CCI, Charlson Comorbidity Index; ICI, immune checkpoint inhibitor; LDH, lactate dehydrogenase; NLR, neutrophil-to-lymphocyte ratio; PD-1, programmed cell death protein 1; PD-L1, programmed death-ligand 1; CTLA-4, cytotoxic T-lymphocyte-associated protein 4.

**Supplementary Table S7. Univariate analysis of factors influencing overall survival among patients with HPD diagnosis.**

| **Variables^a^** | **Univariate analysis, HR (95% CI)** | ***P* value^b^** |
| --- | --- | --- |
| Male sex | 2.67 (1.14-3.66) | 0.030 |
| Age ≥65 | 1.36 (0.80-2.31) | 0.280 |
| ECOG PS ≥ 2 | 1.15 (0.53-2.31) | 0.780 |
| CCI >10 | 1.84 (0.94-3.56) | 0.077 |
| Driver mutation | 0.879 (0.33-1.88) | 0.830 |
| Metastatic disease | 1.35 (0.58-3.18) | 0.456 |
| Brain metastasis | 2.33 (1.11-54.88) | 0.037 |
| Liver metastasis | 1.42 (0.83-2.46) | 0.30 |
| Concomitant chemotherapy | 0.69 (0.30-1.63) | 0.540 |
| Line of ICI administration | 1.00 (0.69-1.60) | 0.830 |
| Type of ICI^c^ | 0.69 (0.32-1.47) | 0.367 |
| Adverse events | 0.63 (0.30-1.31) | 0.266 |
| Albumin ≤30 g/L | 1.63 (0.79-3.12) | 0.190 |
| Metastatic sites >2 | 1.91 (1.02-3.51) | 0.015 |
| LDH >300 UI/L | 1.13 (0.58-2.19) | 0.70 |
| NLR ≥4 | 1.47 (0.84-2.54) | 0.174 |

^a^ Univariable analyses were conducted in the overall HPD cohort; the number of evaluable patients varied according to data availability for each variable.

^b^ Only variables with a *P* value < 0.2 were included in the multivariate analysis.

^c^ Refers to the following categories: anti-PD-1 inhibitors, anti-PD-L1 inhibitors, or combination therapy consisting of anti-CTLA-4 plus anti-PD-1/PD-L1 inhibitors.

Abbreviations: HPD, hyperprogressive disease; CI, confidence interval; HR, hazard ratio; NE, not evaluated; ECOG PS, Eastern Cooperative Oncology Group Performance Status; CCI, Charlson Comorbidity Index; ICI, immune checkpoint inhibitor; LDH, lactate dehydrogenase; NLR, neutrophil-to-lymphocyte ratio; PD-1, programmed cell death protein 1; PD-L1, programmed death-ligand 1; CTLA-4, cytotoxic T-lymphocyte-associated protein 4.

**Supplementary Figures**

**
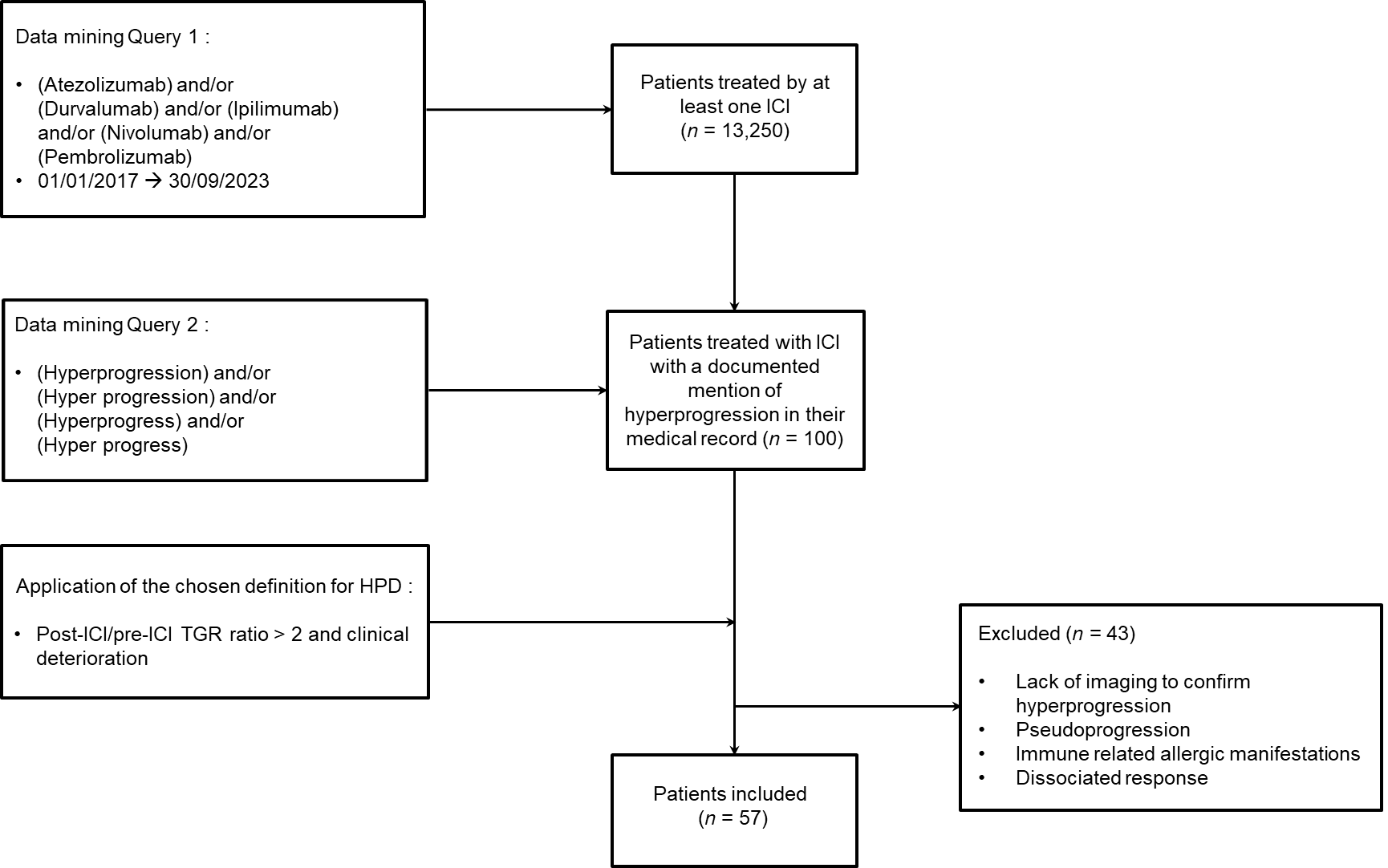
**

**Supplementary Figure S1. Workflow of the HYPERPROG study, characterizing HPD in a real-life setting**. Abbreviations: ICI, immune-checkpoint inhibitor; HPD, hyperprogressive disease; TGR, tumor growth rate

**
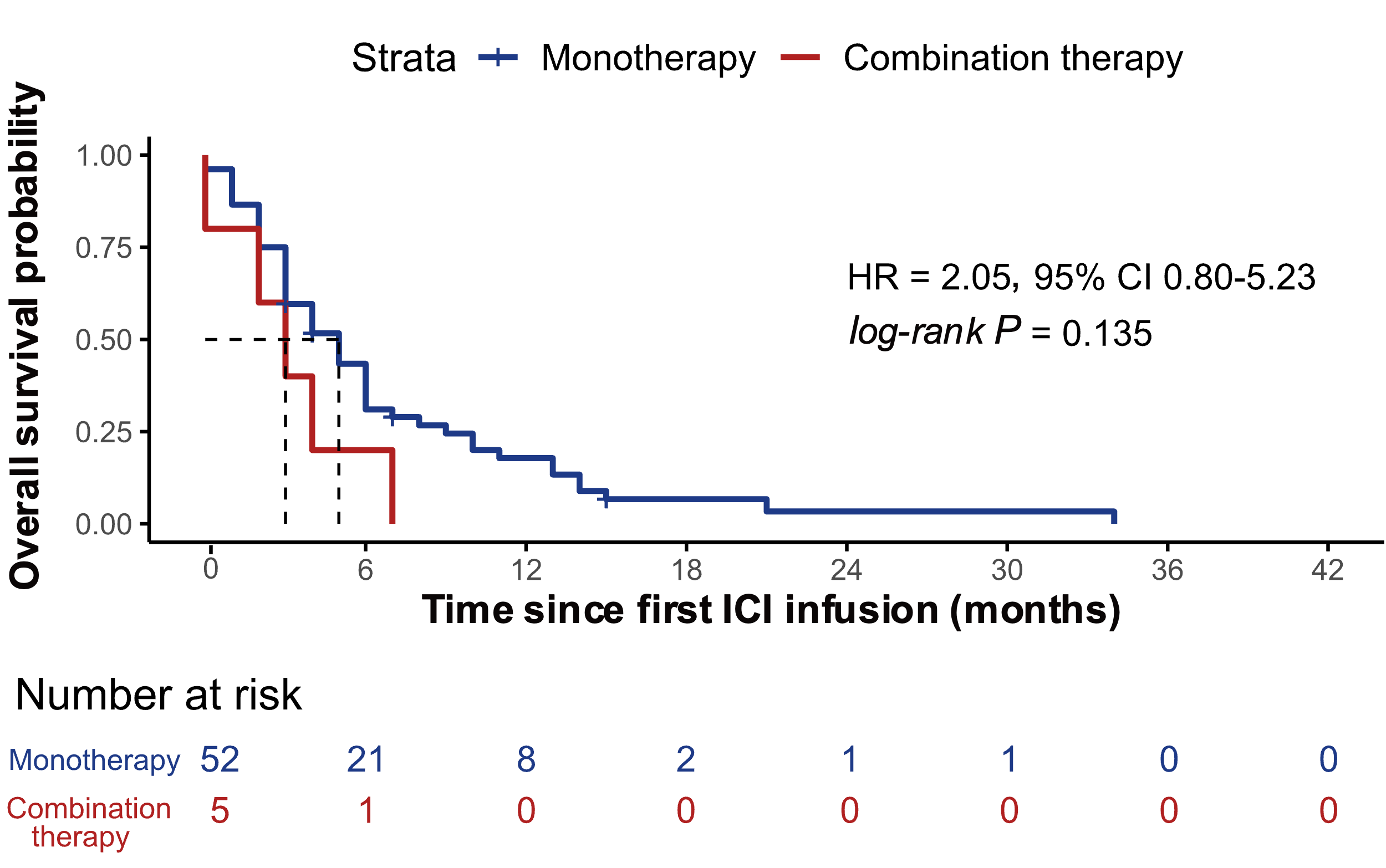
**

**Supplementary Figure S2. Overall survival in patients with hyperprogressive disease according to ICI regimen: combination therapy (anti-CTLA-4 plus anti-PD-1) versus monotherapy (anti-PD-1 or anti-PD-L1).** Abbreviations: ICI, immune checkpoint inhibitors; HR, hazard ratio; CI, confidence interval; CTLA-4, cytotoxic T-lymphocyte-associated protein 4; PD-1, programmed cell death protein 1; PD-L1, programmed death-ligand 1.

**
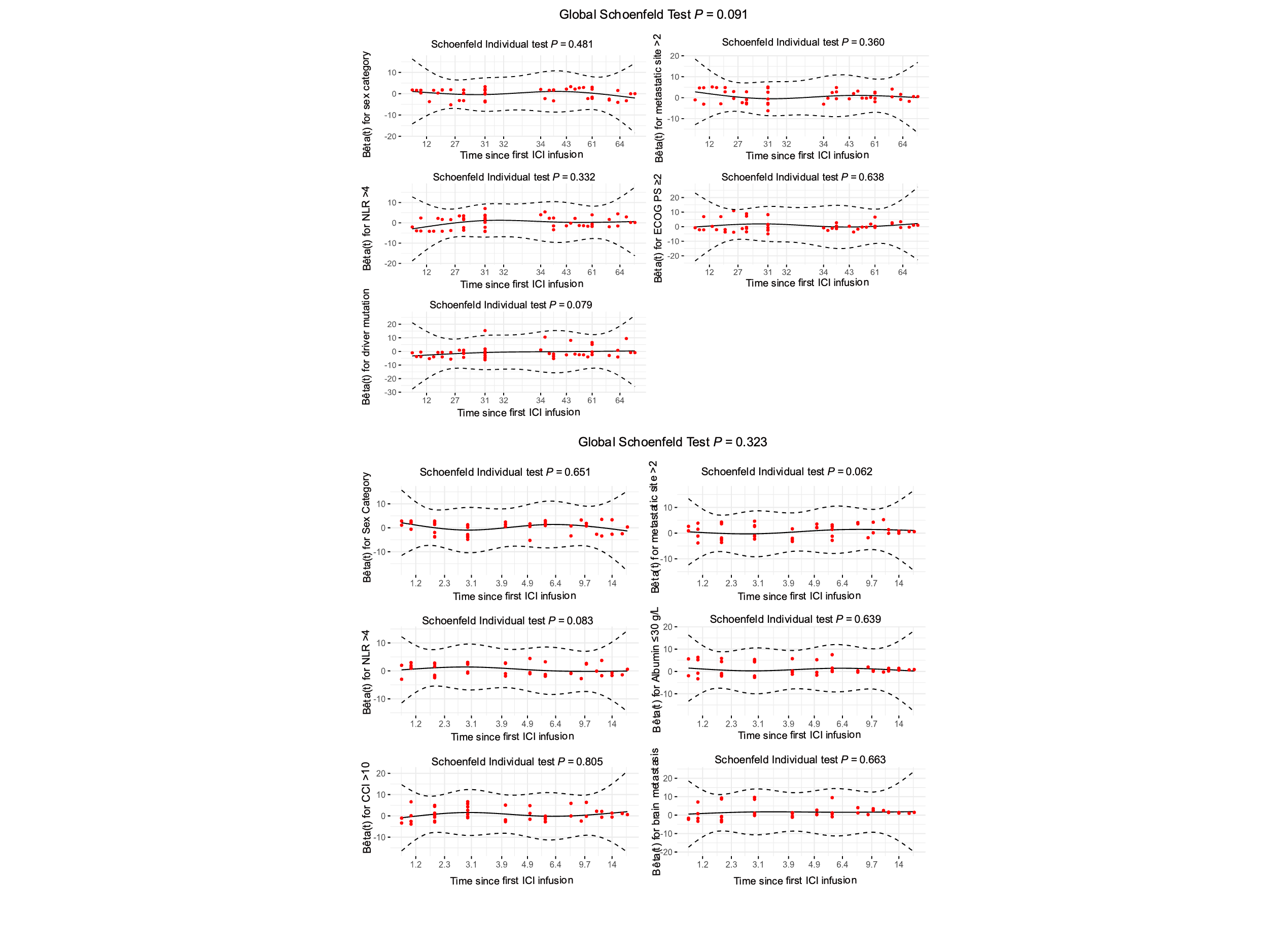
**

**Supplementary Figure S3**. **Schoenfeld test for selected variables in multivariate analysis (Time to HPD and OS).** Abbreviations: ECOG PS, Eastern Cooperative Oncology Group Performance Status; NLR, neutrophil-to-lymphocyte ratio; Charlson, Charlson Comorbidity Index; metastatic site >2, more than two metastatic sites; HPD, hyperprogressive disease; OS, overall survival.

**References**

1. Guerin J, Nahid A, Tassy L, Deloger M, Bocquet F, Thezenas S, et al. Consore: A Powerful Federated Data Mining Tool Driving a French Research Network to Accelerate Cancer Research. Int J Environ Res Public Health. 21. Switzerland2024.

2. Champiat S, Dercle L, Ammari S, Massard C, Hollebecque A, Postel-Vinay S, et al. Hyperprogressive Disease Is a New Pattern of Progression in Cancer Patients Treated by Anti-PD-1/PD-L1. Clin Cancer Res. 23. United States2017. p. 1920–8.

3. Facchinetti F, Lo Russo G, Tiseo M, Garassino MC, Ferrara R. How to recognize and manage hyper-progression and pseudo-progression during immune checkpoint blockade in non-small cell lung cancer. Precision Cancer Medicine. 2019;2:35.

4. Liu J, Wu Q, Wu S, Xie X. Investigation on potential biomarkers of hyperprogressive disease (HPD) triggered by immune checkpoint inhibitors (ICIs). Clin Transl Oncol. 23. Italy2021. p. 1782–93.

5. Kim JY, Lee KH, Kang J, Borcoman E, Saada-Bouzid E, Kronbichler A, et al. Hyperprogressive Disease during Anti-PD-1 (PDCD1) / PD-L1 (CD274) Therapy: A Systematic Review and Meta-Analysis. Cancers (Basel). 11. Switzerland2019.

6. Zhao Z, Bian J, Zhang J, Zhang T, Lu X. Hyperprogressive disease in patients suffering from solid malignancies treated by immune checkpoint inhibitors: A systematic review and meta-analysis. Front Oncol. 2022;12:843707.

7. Charlson ME, Pompei P, Ales KL, MacKenzie CR. A new method of classifying prognostic comorbidity in longitudinal studies: development and validation. J Chronic Dis. 1987;40(5):373–83.

8. Bagni K, Chen IM, Johansen AZ, Dehlendorff C, Jensen BV, Hansen CP, et al. Prognostic impact of Charlson's Age-Comorbidity Index and other risk factors in patients with pancreatic cancer. Eur J Cancer Care (Engl). 2020;29(3):e13219.

9. Friedlaender A, Banna GL, Buffoni L, Addeo A. Poor-Performance Status Assessment of Patients with Non-small Cell Lung Cancer Remains Vague and Blurred in the Immunotherapy Era. Curr Oncol Rep. 21. United States2019. p. 107.

10. Zer A, Sung MR, Walia P, Khoja L, Maganti M, Labbe C, et al. Correlation of Neutrophil to Lymphocyte Ratio and Absolute Neutrophil Count With Outcomes With PD-1 Axis Inhibitors in Patients With Advanced Non-Small-Cell Lung Cancer. Clin Lung Cancer. 2018;19(5):426–34 e1.

11. Driscoll JJ, Rixe O. Overall survival: still the gold standard: why overall survival remains the definitive end point in cancer clinical trials. Cancer J. 15. United States2009. p. 401–5.

12. Beaufils M, Amodru V, Tejeda M, Boher JM, Zemmour C, Chanez B, et al. Dysthyroidism during immune checkpoint inhibitors is associated with improved overall survival in adult cancers: data mining of 1385 electronic patient records. J Immunother Cancer. 11. England2023.
